# Exosomes from BM-MSCs increase the population of CSCs via transfer of miR-142-3p

**DOI:** 10.1038/s41416-018-0254-z

**Published:** 2018-09-17

**Authors:** Hongdan Li, Feng Li

**Affiliations:** Department of Cell Biology, Key Laboratory of Cell Biology, National Health Commission of the PRC, and Key Laboratory of Medical Cell Biology, Ministry of Education of the PRC, China Medical University, No. 77, Puhe Road, Shenyang North New Area, 110122 Shenyang, Liaoning China

**Keywords:** Cancer stem cells, Cancer microenvironment

## Abstract

**Background:**

Bone marrow-derived mesenchymal stem/stromal cells (BM-MSCs) are progenitor cells shown to migrate to the tumour and participate in the tumour microenvironment. BM-MSCs play important roles in tumour processes through the release of cytokines or exosomes; however, how BM-MSCs influence the stemness of CSCs in colon cancer cells remains poorly understood.

**Methods:**

We isolated exosomes from BM-MSCs and used these exosomes to treat colon cancer cells (HCT-116, HT-29 and SW-480). We compared stemness traits of colon CSCs by cell surface marker (CD133 and Lgr5) and functional assays, such as chemoresistance, colony formation, cell adhesion, invasion and tumour-formation assay. We performed a microRNA array to investigate the differences in exosomal microRNA expression between colon cancer cells, BM-MSCs and co-cultured cells and performed functional and molecular analysis of the gene targets.

**Results:**

In this study, we found that BM-MSC-derived exosomes contained distinct microRNAs, including miR-142-3p, which in turn increased the population of CSCs in colon cancer cells. Depriving miR-142-3p from BM-MSC-derived exosomes clearly decreased the population of colon CSCs. Mechanistically, Numb was found to be the target gene of miR-142-3p, and miR-142-3p promoted the Notch signalling pathway by downregulating Numb.

**Conclusions:**

Our findings indicate that BM-MSC-derived exosomes promote colon cancer stem cell-like traits via miR-142-3p.

## Introduction

Colon cancer is among the three leading causes of cancer-related deaths worldwide,^1^ and even though many targeted drugs have been developed to treat colon cancer, surgical removal and chemo/radiation therapy remains standard of care.^2^ Moreover, colon cancer patients often develop metastatic disease or recur years after comprehensive therapeutic regimen of the primary tumour. The patients are asymptomatic because the disseminated cells appear to become dormant and are undetectable. The dormant cancer cells have been attributed to cancer stem cells. Colon cancer stem cells (CSC)^3^ are believed to be resistant to chemo/radiation therapy and a major cause of relapse.^4,5^.  However, since CSCs is similar to normal stem cells, developing treatments for the dormant CSCs are arduous. Therefore, the next major advance in this area is to find a deeper mechanism of events which promote CSCs phenotype in the colon tumour microenvironment.

The colon tumour microenvironment, as with other tumours, contains many factors and cell types, such as immune cells, stromal cells and endothelial cells, that influence the tumour.^6^ Nowadays, there is an accumulating amount of evidence demonstrating that bone marrow-derived mesenchymal stem cells (BM-MSCs) could also migrate to primary tumours and participate in the tumour microenvironment, contribute to tumour stroma formation and affect tumour cell activity.^7,8^ The interaction between BM-MSCs and tumour cells may occur through many manners. BM-MSCs could secrete a variety of cytokines in the tumour milieu.^9^ MSCs could also cause a direct effect through intercellular signalling via physical contacts with neighbouring tumour cells and ultimately determine the fate of the tumour growth kinetics. In addition to this, some studies^10–14^ suggest that MSCs have special delivery systems requiring exosomes. Exosomes are small (50–100 nm) vesicles of endocytic origin that are released in the extracellular milieu by several cell types. It was recently reported that exosomes also contain messenger RNA (mRNA) and microRNA that are transferred to target cancer cells, where they can be translated or mediate RNA silencing.^15–18^ Together, BM-MSCs play vital roles in many tumour processes. However, how BM-MSC-derived exosomes influence tumour stemness is unclear.

Here we will demonstrate that BM-MSC-derived exosomes promote colon CSC-like traits and exosomal miR-142-3p plays vital roles in this process. Further, we discover that the target gene of miR-142-3p, Numb, is responsible for this process.

## Materials and methods

### Isolation and characterisation of human mesenchymal stem cells

Bone marrow cells were isolated from the femoral head after informed consent from patients undergoing hip-replacement surgery. The marrow were mixed with culture medium (MesenPRO RS™ Medium, Gibco, 12746–012) and isolated by the h-BM-MSC Isolation Kit (TBD). The collected cells were plated in tissue culture flasks without further interference for 2–3 days. The culture medium was depleted by successive changes of culture medium (MesenPRO RS™ Medium, Gibco, 12746–012). A confluent monolayer of cells was observed 7 days following initial plating. Human BM-MSCs were characterised by the BD Human MSC Analysis Kit (BD 562245).

### Exosome isolation

Before exosome collection, the BM-MSCs were cultured in culture media containing centrifuged foetal bovine serum (FBS), which was used to remove FBS-derived exosomes. During 24–48 h, the culture medium was collected and prepared for exosome collection. Exosomes were collected from the medium of 50 ml human BM-MSC cells. The culture media was placed on ice and centrifuged at 800 × *g* for 10 min to sediment the cells and subsequently centrifuged at 12,000 × *g* for 20 min to remove cellular debris. Exosomes were separated from the supernatant by centrifugation at 100,000 × *g* for 2 h. The exosome pellet was washed once in a large volume of phosphate-buffered saline (PBS) and re-suspended in 100 μl of PBS (exosome fraction). For nanoparticle tracking analysis, the size of the exosomes was characterised by dynamic light scattering (Zetasizer Nano ZS, Malvern Instruments, Malvern, UK).

### Cell culture and treatment

Human colon cancer cell lines HCT-116, HT-29 and SW-480 were purchased from Genechem Biotechnology Company (The cell lines of Genechem Biotechnology Company were purchased from ATCC). All the cells were maintained in Dulbecco’s modified Eagle’s medium with 10% FBS (Life Technologies, NY) and 1% antibiotic–antimycotic solution (Life Technologies, NY).

For the exosome treatment, colon cancer cells (HCT-116, HT-29 and SW-480) were treated with 10 μg/ml of BM-MSC-derived exosomes or control PBS q.o.d for 1–2 weeks. For example, at 1, 3, 5, 7, 9.

### Electron microscopy

Exosomes were adsorbed for 10 min to a carbon-coated grid rendered hydrophilic and fixed for 20 min with 4% paraformaldehyde. The excess liquid was removed with a filter paper, and samples were stained with 1% uranyl acetate for 30 s. After excess uranyl formate was removed with a filter paper, grids were examined, and images were recorded by transmission electron microscope (Japan, Hitachi 7650). For immunogold labelling, carbon-coated grids containing exosomes were fixed with 2% paraformaldehyde in 0.1 M phosphate buffer (pH 7.4), then processed for 20 mM Glycine washing and immunogold labelling using anti-CD63, anti-CD81 and Rab 5 A antibodies overnight at 4 °C and the second antibody with 10- or 15-nm gold particles for 1 h at room temperature. The grids were observed at 80 kV with a Transmission electron microscope, and images were recorded with an AMT 2k CCD camera.

### MiRNA array analyses

The exosomes derived from human BM-MSCs, colon cancer cells and co-cultured human BM-MSCs/colon cancer cells were all analysed by microRNA array. The Human microRNA Array was run by Kangchen Bio-tech Incorporated company. The levels of selected microRNAs were determined using quantitative real-time PCR with SYBR and conducted using a Stratagene system (Mx3000P). MicroRNAs that were expressed in exosomes from the BMSC group (+), highly expressed in the co-cultured cells group (+++) and had low expression in the colon cancer cell group (+/−) were selected. Based on this principle, many microRNAs were excluded and >3-fold microRNAs were selected.

### Dual-luciferase reporter assay

Plasmids were used that encoded a portion of the 3′-untranslated region (3′UTR) of NUMB linked to the firefly luciferase protein. Firefly luciferase constructs were co-transfected with Renilla luciferase vector control (TK) into HCT116 cells. Where indicated, HCT116 cells were stably expressing miR-142-3p. Twenty-four hours after co-transfection with the 3′UTR of the target gene and TK (ratio 1:10), HCT-116 cells were detached, washed and dissolved in passive lysis solution for 15 min at room temperature. Luciferase activities were measured consecutively (Dual-Luciferase Assay; Promega), and the relative luciferase activity was assessed: (firefly activity)/(Renilla activity).

### Flow cytometric (FCM) analysis

The CD133-APC antibody (Miltenyi Biotec, 130-090-826) and Lgr5-PE antibody (Miltenyi Biotec, 130-112-437) staining was performed according to the manufacturer’s instructions, followed by FCM analysis using BD LSRFortessa.

### Transfections and stable cell lines

Stable cell lines overexpressing miR-142-3p and control were generated from parental HCT-116, HT-29 and SW-480 cells. MiR-142-3p and control lentivirus were purchased from Genechem Biotechnology Company. MiR-142-3p or control lentivirus were transfected into different cell lines according to the manufacturer’s recommendations.

### Plasmids transfection

The Numb 3′UTR containing the miR-142-3p-binding site or miR-142-3p-binding site with mutated fragments were cloned into a pGL3-Control vector. The short hairpin RNA against Numb or Numb wild-type expression plasmids were transfected into different cells using lipofectamine 2000 (Life Technologies), according to the manufacturer’s recommendations. After transfection, the RNA or protein were extracted after 48 h, respectively.

### MTT (3-[4,5-dimethylthiazol-2-yl]-2,5 diphenyl tetrazolium bromide) assay

In vitro drug resistance was assessed using tetrazolium salt MTT assay. The colon cancer cells treated with exosomes for 1 week or colon cancer cells with stable miR-142-3p expression were collected and re-plated into a 96-well plate (10,000 cells per well). Doxorubicin was added into the cells at different concentrations for 48 h. Assays were performed by incubating each well with 20 μl MTT substrate for 4 h; the medium was removed and 100 μl dimethyl sulphoxide (DMSO) was added. Plates were read at a wavelength of 490 nm, with reported optical density values normalised to blank wells containing DMSO only.

### Colony-formation assay

For the colony-formation assay, 1000 cells were plated per 6-well plate, respectively. After 10 days, colonies were stained by 4% crystal violet. For the exosome treatment, colon cancer cells (HCT-116, HT-29 and SW-480) were treated with 10 μg/ml BM-MSC-derived exosomes or control PBS at days 1, 3, 5, 7, 9, 11 and 13 for 2 weeks.

### Cell invasion assay

Transwell cell invasion assays were performed using Boyden chambers with a polycarbonate Nucleopore membrane. Precoated filters (6.5 mm in diameter, 8 µm pore size, Matrigel 100 µg/cm^2^) were rehydrated with 100 µl medium. In all, 1 × 10^5^ cells in 100 ml serum-free Dulbecco’s modified Eagle’s medium supplemented with 0.1% bovine serum albumin (BSA) were placed in the upper part of each chamber and the lower compartments were filled with 600 µl Dulbecco’s modified Eagle’s medium containing 10% serum as described previously. For the exosome treatment, colon cancer cells (HCT-116, HT-29 and SW-480) were treated with 10 μg/ml of BM-MSC-derived exosomes or control PBS at day 1, 3, 5 and 7 for 1 week.

### Adhesion assay

The binding ability of tumour cells with fibronectin was detected using a cell adhesion assay. Briefly, pretreated cells (10^4^ each well) were trypsinised, re-plated on fibronectin-coated coverslips (10 μg/ml) and incubated at 37 °C for 1 h. Following incubation, non-adherent cells were removed by washing 3 times with PBS, adherent cells were fixed with 4% paraformaldehyde solution, stained with crystal violet (1%) and dissolved by 1% sodium dodecyl sulphate (SDS). Absorbances at 595 nm were determined using a microplate reader. For the exosome treatment, colon cancer cells (HCT-116, HT-29 and SW-480) were treated with 10 μg/ml of BM-MSC-derived exosomes or control PBS at days 1, 3, 5 and 7 for 1 week.

### Immunocytochemistry

Cells were fixed and tissue sections were de-waxed and re-hydrated, then blocked with 1% BSA for 1 h and incubated 4 °C overnight in the dark, with primary antibodies combined with Alexa Fluor 488 (green) or 564 (red) reagents: E-cadherin (cat. 14472, 1:100 dilution, Cell Signalling Technology), N-cadherin (cat. ab98952, 1:100 dilution, Abcam), Vimentin (cat. ab92547, 1:100 dilution, Abcam), and anti-mouse alpha smooth muscle actin (α-SMA; cat. ab5694, 1:20 dilution, Abcam). Antibodies were diluted in PBST (PBS plus 0.2% Triton X-100). The cells and tissue sections were post-fixed with 10% formalin for 10 min followed by processing with 4,6-diamidino-2-phenylindole. Images were acquired with an Olympus FV10i confocal microscope.

### Quantitative real-time PCR assays

microRNAs were isolated from total RNA and reverse transcribed. Large RNAs were isolated with TRIzol reagent (Invitrogen) and reverse transcribed (C10710, miDETECT A Track™ miRNA qRT-PCR Starter Kit, Guangzhou RiboBio Co., Ltd.). cDNAs were amplified by reverse transcriptase–PCR. Expression assays were used to quantify the levels of different RNAs as follows: hsa-miR-142-3p, U6, CD44, Oct-4, SOX2, KLF, Lin28, Lgr5, Bmi1, Numb, Actin, and glyceraldehyde 3-phosphate dehydrogenase (GAPDH). Quantitative PCR was conducted in triplicate at 95 °C for 10 min, followed by 40 cycles of 95 °C for 15 s and 60 °C for 60 s (7300 Fast Real-Time PCR System; Stratagen). Cycle thresholds were normalised to an internal control: U6 or 18S rRNA for precursor of miR and GAPDH or Actin for mRNA assays. The amount of RNA was calculated using the 2^−^^ΔΔCT^ method; the level of expression of an RNA was normalised to the adapted internal control (denoted “relative expression”) and, where appropriate, to the level of expression at 1 min of coculture (denoted “fold change”). The primers of miR-142-3p,18S rRNA and U6 were synthesised in RiboBio Co., Ltd. The other primers used in this study can be found in Supplementary Table 1.

### Orthotopic tumour growth in BALB/c Nu Nu mice

In situ model: 200,000 colon cancer cells (HCT-116, HT-29 and SW-480) were seeded into 25 cm^2^ culture bottles, and 500 μg of BM-MSCs-derived exosomes or the same amount of PBS was added to each bottle on days 1 and 4 and incubated for 7 days before being injected into the intestinal wall of BALB/C nu/nu mice. Additionally, miR-142-3p overexpression colon cancer cells were also implanted into the intestinal wall of BALB/c Nu/Nu mice. The control colon cancer cells, exosome-treated colon cancer cells or miR-142-3p overexpression colon cancer cells were injected into the intestinal wall of Balb/c nu/nu mice, and 5–7-week-old female Balb/c nu/nu mice received intestinal wall 100 μl injections of PBS containing 1 × 10^6^ colon cancer cells that had been cultured with BM-MSC-derived exosomes or PBS for 30 days. The tumours (primary or secondary) were excised and subjected to pathological examination. All experimental protocols involving animals were performed with IACUC-approved protocols. The BALB/c nu/nu mice were purchased from Beijing Vital River Laboratory Animal Technology Co., Ltd.

Subcutaneous model: The 5-week-old BALB/c-nu mice were randomly divided into three groups (*n* = 5 per group). To explore how BM-MSC-derived exosomes influence the progression of colon cancer cells in vivo, exosomes from BM-MSCs or the same volume of PBS was added to colon cancer cells at days 1 and 4 and incubated for 7 days, before being injected into the intestinal wall of BALB/C nu/nu mice. Colon cancer cells (1 × 10^5^, 1 × 10^4^, 1 × 10^3^) were inoculated subcutaneously into the right flank of the nude mice. The mice were sacrificed 35 days after inoculation and the tumours were analysed by tumour weight.

### Western blot analyses

Cells or exosomes were lysed in RIPA buffer with protease inhibitors. Lysates separated on 10% SDS-polyacrylamide gel and proteins were transferred to polyvinylidene difluoride membranes. The membranes were incubated with 5% milk for 1 h and probed overnight at 4 °C with antibodies targeting CD63 (cat. AP6631B, 1:1000 dilution, Abgent), CD81 (cat. 556019, 1:1000 dilution, BD), Rab5a (cat. NB120-13253, 1:1000 dilution, Abgent), CD9 (cat. 13174, 1:1000 dilution, Cell Signaling Technology), HSP70 (cat. 4876, 1:1000 dilution, Cell Signaling Technology), Cytochrome *c* (cat. 4272, 1:1000 dilution, Cell Signaling Technology), Numb (cat. 2756, 1:1000 dilution, Cell Signaling Technology) and β-Actin (cat.4970, 1:5000 dilution, Cell Signaling Technology). After washing, blots were incubated with peroxidase-conjugated anti-rabbit IgG horseradish peroxidase secondary antibodies for 1 h at room temperature. Protein expression was detected with Pierce™ ECL Western Blotting Substrate (cat. 32106, Thermo Scientific, Rockford).

### Statistical analyses

The data presented in bar graphs are means ± SD of at least three independent experiments. The statistical analyses were performed with Student’s *t*- test. *P* < 0.05 was considered to be statistically significant.

### Study approval

The use of human colon cancer tissue specimens was evaluated and approved by the Ethical Committee of Surgical Oncology of the First Affiliated Hospital of China Medical University, and written informed consent was obtained from all participants or their appropriate surrogates. All the experimental procedures were approved by the Institutional Animal Care and Use Committee of the China Medical University in accordance with guidelines set forth by the University for Animal Research.

## Results

### Characterisation of MSCs derived from human bone marrow

**Fig. 1:**
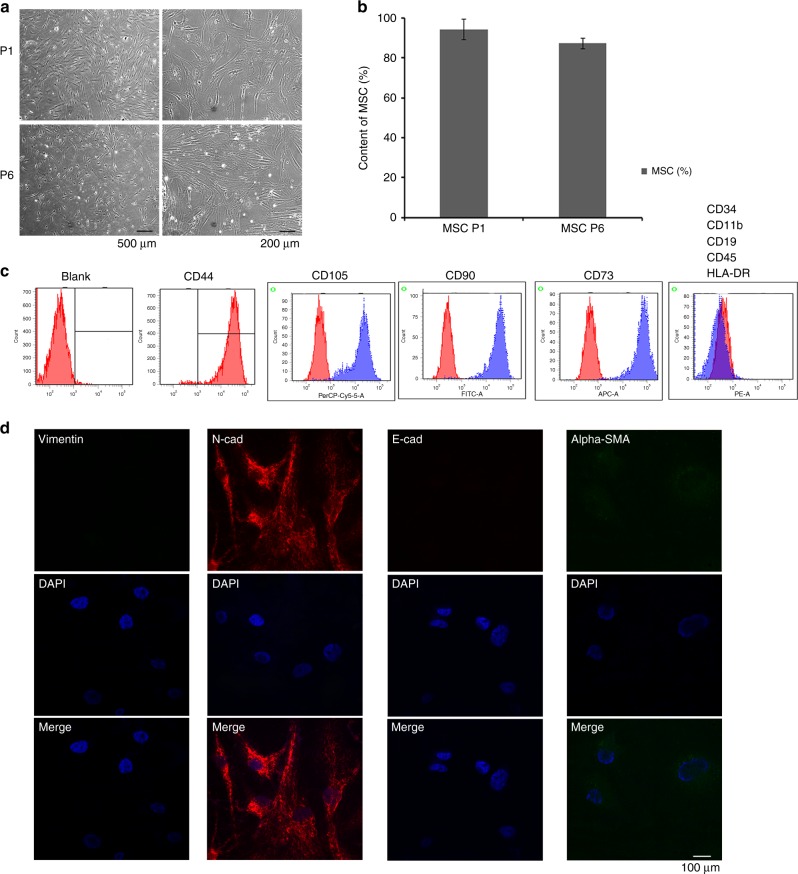
Characteristics of MSCs derived from human bone marrow. **a** Representative morphology of BM-MSCs. Magnification: ×40 (left) and ×100 (right). **b** The analysis of stemness in P1 and P6 BM-MSC. **c** Flow cytometric analysis showed BM-MSCs were positive for mesenchymal lineage markers (CD44, CD73, CD90 and CD105), negative for haematopoietic and endothelial markers (CD34, CD11b, CD19, CD45) and negative for HLA-DR. **d** Immunofluorescence staining of BM-MSCs showed that they were positive for mesenchymal markers of Vimentin (green), α-SMA (green) and N-cadherin (red) and negative for epithelial markers of E-cadherin; ×400, scale bar = 100 μm

Human BM-MSCs were isolated from human bone marrow cells obtained from healthy individuals by FCM (CD44, CD90, CD73, CD105). The purity of BM-MSCs as evaluated by flow cytometer was >95% (Passage 1). The adherent cells showed homogenous fibroblastic morphology (Fig. 1a–c). At passage 6 (P6), BM-MSCs possessed uniform surface markers and were positive for mesenchymal markers (CD44, CD73, CD90, and CD105), negative for haematopoietic and endothelial markers (CD34, CD11b, CD19, CD45) and HLA-DR, shown in Fig. 1b, and the purity of P6 BM-MSCs was ~87.4%. Furthermore, immunofluorescence staining demonstrated that BM-MSCs were positive for N-cadherin and weak positive for α-SMA and negative for E-cadherin and Vimentin (Fig. 1d).

### Isolation and identification of BM-MSC-derived exosomes

**Fig. 2:**
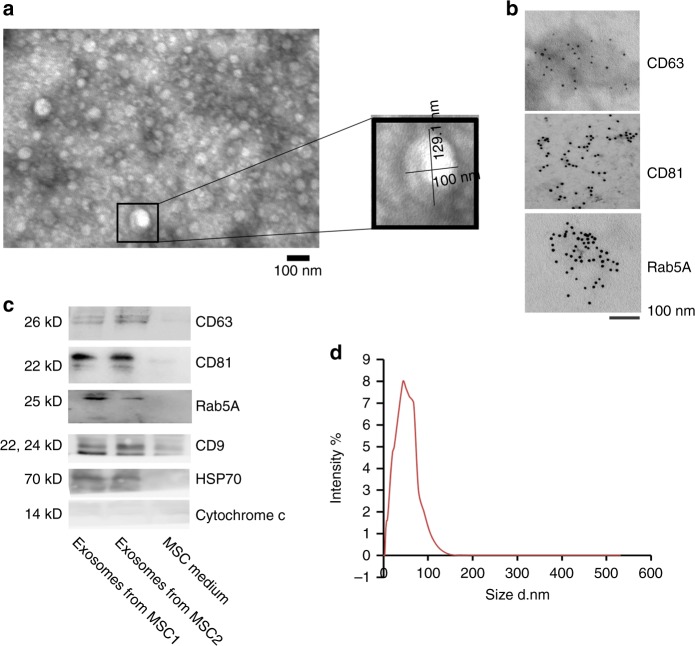
Characterisation of BM-MSC-derived exosomes. **a** Electron microscopy of BM-MSCs-derived exosomes, showing small vesicles of 129.1nm × 100 nm. **b** Exosomes from BM-MSCs were immunogold-labelled with anti-CD63, anti-CD81 and Rab5A antibodies. Scale bar = 100 nm. **c** Western blot showed the expression of CD63, CD81, Rab5A, CD9, HSP70 and Cytochrome *c* in BM-MSCs-derived exosomes (*n* = 2) and BM-MSCs conditional medium (*n* = 1) proteins. **d** Size distributions of BM-MSC-derived exosomes by nanoparticle tracking analysis (NTA)

To identify whether the exosomes from BM-MSCs play roles on the CSC traits of colon cancer, we isolated the exosomes derived from BM-MSCs by standard ultracentrifugation. Using transmission electron microscopy, we determined that BM-MSC-derived exosomes were about 50–130 nm in width and physically homogeneous (Fig. 2a). To detect the size distribution of exosomes in detail, we demonstrated that the size of BM-MSC-derived exosomes ranged from 3.6 to 140 nm by particle size analyser (Fig. 2d). The exosomal expression of CD63, CD81 and Rab5A surface antigens commonly used as exosomal markers^19,20^ were subsequently evaluated and confirmed using immunogold labelling. Through immuno-electron microscope, we confirmed that the exosomes were positive for the known exosome markers CD63, CD81 and RAb5A (Fig. 2b, c). To further identify the exosomes derived from BM-MSCs, we detected the expression of CD63, CD81, Rab5A, CD9, HSP 70 and Cytochrome *c*. The exosomes were positive for the expression of CD63, CD81, Rab5A, CD9 and HSP 70 and negative for the expression of Cytochrome *c* by western blot (Fig. 2c). We excluded exosomes mixed with apoptotic bodies.

### BM-MSC-derived exosomes increase the population of colon CSCs

**Fig. 3:**
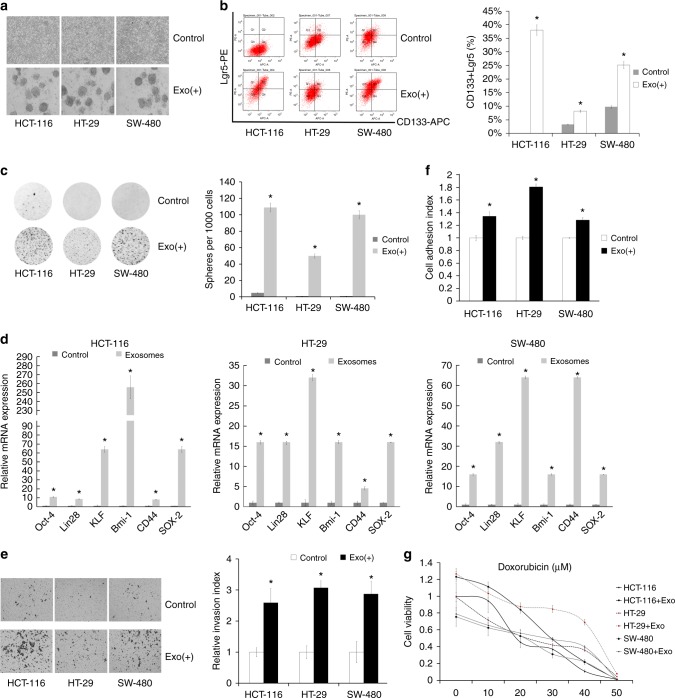
BM-MSC-derived exosomes promote colon cancer stem cell-like traits. **a** After BM-MSC-derived exosome treatment, colon cancer cells formed cancer stem-like spheres. **b** Colon cancer cells were co-cultured with isolated exosomes from BM-MSCs or treated by PBS, and the CD133- and Lgr5-positive cell population was determined. Quantification is shown at right. **c** Colony-formation assay showed that the colony-forming ability was enhanced upon BM-MSC-derived exosomes. **d** Stem cell markers (OCT4, Lin28, KLF, Bmi-1, CD44 and SOX2) in colon cancer cells were increased upon treatment with BM-MSC-derived exosomes. *P < 0.05. **e** Cell invasion by colon cancer cells cultured with or without exosomes normalised to the invasion index of cells cultured without exosomes. Data are means ± SD (*n* = 3; *P < 0.05, cells with exosomes vs. cells without exosomes Student’s *t* test). **f** Cell adhesion by colon cancer cells cultured with or without exosomes normalised to the invasion index of cells cultured without exosomes. Data are means ± SD (*n* = 3; *P < 0.05, cells with exosomes vs. cells without exosomes, Student’s *t*- test). **g** Drug resistance assay examined colon cancer cells treated with doxorubicin alone or in the presence of exosomes

To test whether BM-MSC-derived exosomes could promote stemness in colon cancer cells, we treated colon cancer cells (HCT-116, HT-29, SW-480) with exosomes derived from the conditional medium from BM-MSCs. After 10–15 days of incubation with exosomes q.o.d, the colon cancer cells formed cancer stem-like spheres (Fig. 3a). We performed FCM and found that the exosome treatment increased the expression levels of CD133^+^ and Lgr5^+^ colon cancer cells (Fig. 3b). We performed the colony-formation assay and colony numbers were increased when colon cancer cells were treated with exosomes, compared with control (colon cancer cells alone) (Fig. 3c). We also found increased expression of stem cell markers, including OCT4, Lin28, KLF, Bmi-1, CD44 and SOX2, in colon cancer cells upon treatment with BM-MSCs-derived exosomes (Fig. 3d).

To identify whether the BM-MSC-derived exosome-induced CSCs exhibited the same characteristics as CSCs, we performed several experiments to explore the characteristics of the colon CSCs induced by BM-MSC-derived exosomes and PBS as a control. We examined cell invasion and tumourigenicity in colon cancer cells and found that the colon cancer cells treated with exosomes had significantly increased cell invasion and tumourigenicity (Fig. 3e and Supplementary Fig. S6). As shown in Fig. S6A–B, tumours in the exosomes from the BM-MSC group were bigger than tumours in the negative control group or Lentivirus-transfected group, following implantation of 1 × 10^5^ or 1 × 10^4^ cells. Importantly, only exosome-treated cells formed tumours after 1 × 10^3^ cells were implanted (Supplementary Fig. S6C). These results showed that exosomes from BM-MSCs promoted colon cancer cell tumourigenesis in vivo. Additionally, BM-MSC-derived exosomes also promoted cell adhesion (Fig. 3f) compared with those cultured alone. Colon cancer cells treated with exosomes also exhibited greater drug resistance to doxorubicin, compared with control colon cancer cells (Fig. 3g). These findings suggest that the exosomes derived from BM-MSCs play critical roles in promoting the stemness of colon cancer cells.

### Exosomal miR-142-3p promotes a stem cell-like phenotype of colon cancer cells

We next explored which composition of BM-MSC-derived exosomes promoted CSC formation in colon cancer cells. We hypothesised that microRNAs in the BM-MSC-derived exosomes contributed to the increase of colon CSCs. To explore which microRNAs in BM-MSC-derived exosomes promoted the stemness of colon cancer cells, we analysed the microRNA abundance signature in exosomes from BM-MSC, co-cultured medium of BM-MSC and colon cancer cells and the exosomes from colon cancer cells as control. The data were analysed by KangChen Bio-tech Incorporated company. We selected microRNAs that had high expression in exosomes from the BMSC group (+), higher expression in the co-cultured cells group (+++) and low expression in the colon cancer cells group (+/−). Based on this principle, many microRNAs were excluded, and we identified microRNAs with a >3-fold change. The heat map and scatter plot is shown in Figs. S1, 2.

**Fig. 4:**
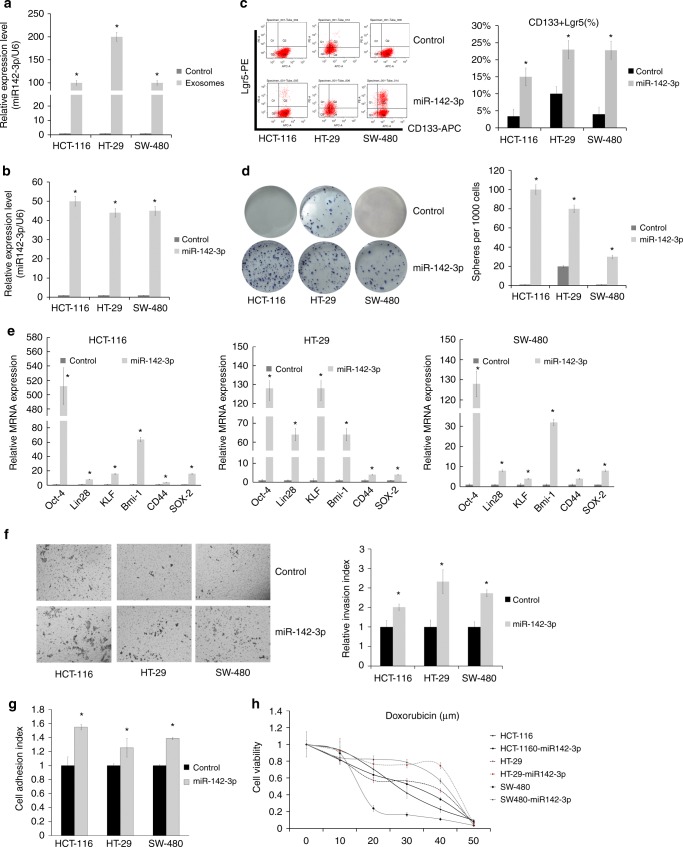
Exosomal microRNA-142-3p promoted CSCs in colon cancer cells. **a** Real-time PCR showed the expression of miR-142-3p in exosomes from colon cancer cells alone or from colon cancer cells treated with BM-MSC-derived exosomes. **b** Real-time PCR showed the expression of exosomal miR-142-3p from colon cancer cells alone or from colon cancer cells transfected with miR-142-3p-Lentivirus. **c** Flow cytometry showed the expression of CD133- and Lgr5-positive cells in colon cancer cells transfected with control Lentivirus or miR-142-3p-Lentivirus. **d** Colony-formation assay showed the colony-forming ability of colon cancer cells transfected with control Lentivirus or miR-142-3p-Lentivirus. *P < 0.05. **e** Real-time PCR of the expression of stem cell markers (OCT4, Lin28, KLF, Bmi-1, CD44 and SOX2) in colon cancer cells transfected with control Lentivirus or miR-142-3p-Lentivirus. *P < 0.05. **f** Cell-invasive ability of colon cancer cells transfected with control Lentivirus or miR-142-3p-Lentivirus, normalised to the invasion index of cells transfected with control Lentivirus. Data are means ± SD (*n* = 3, *P < 0.05). **g** Cell-adhesion ability of colon cancer cells transfected with control Lentivirus or miR-142-3p-Lentivirus. Data are means ± SD (*n* = 3, *P < 0.05). **h** Drug resistance assay showed cell viability of colon cancer cells transfected with Lentivirus or miR-142-3p-Lentivirus treated by doxorubicin. The colon cells used in this part were HCT-116, HT-29 and SW-480

From the microRNA array, we identified 50 microRNAs with a more than three-fold increase in BM-MSCs-derived exosomes (Supplementary Fig. S3). Then we identified the expression of microRNA by real-time PCR and found the correct fold change in exosomes. Supplementary Fig. S4 shows the fold change of miR-142-3p by microRNA array and qPCR. We synthesised mimics for the 50 microRNAs and transfected them to colon cancer cells separately to explore which microRNA could promote the colon cancer cells to form CSC-like spheres. Of these, we selected miR-142-3p from the 50 microRNAs, as this was the only microRNA that promoted the formation of colon cancer stem-like spheres (Supplementary Fig. S5) and the expression of CD133 and Lgr5 by FCM. Using quantitative real-time PCR, we confirmed that the abundance of miR-142-3p from the exosomes of colon cancer cells treated with BM-MSC-derived exosomes was greater than that of colon cancer cells alone (Fig. 4a).

To investigate whether miR-142-3p affected the CSC trait of colon cancer cells, the three colon cancer cells were transfected with miR-142-3p Lentivirus or control Lentivirus. Overexpression of miR-142-3p was confirmed by quantitative real-time PCR (Fig. 4b). After transfection, we found that the three cancer cells transfected with miR-142-3p had a greater proportion of CD133- and Lgr5-positive cells than those transfected with the control (Fig. 4c). In addition, we found increased expression of stem cell markers, including OCT4, Lin28, KLF, Bmi-1, CD44 and SOX2, in colon cancer cells upon treatment of BM-MSC-derived exosomes (Fig. 4d). We also found that the miR-142-3p relatively increased cell invasion and adhesion compared with the control (Fig. 4e, f) and exhibited greater drug resistance to doxorubicin compared with control colon cancer cells (Fig. 4g). These findings indicated that exosomal miR-142-3p was responsible for the increase of human colon CSCs in colon cancer cells.

**Fig. 5:**
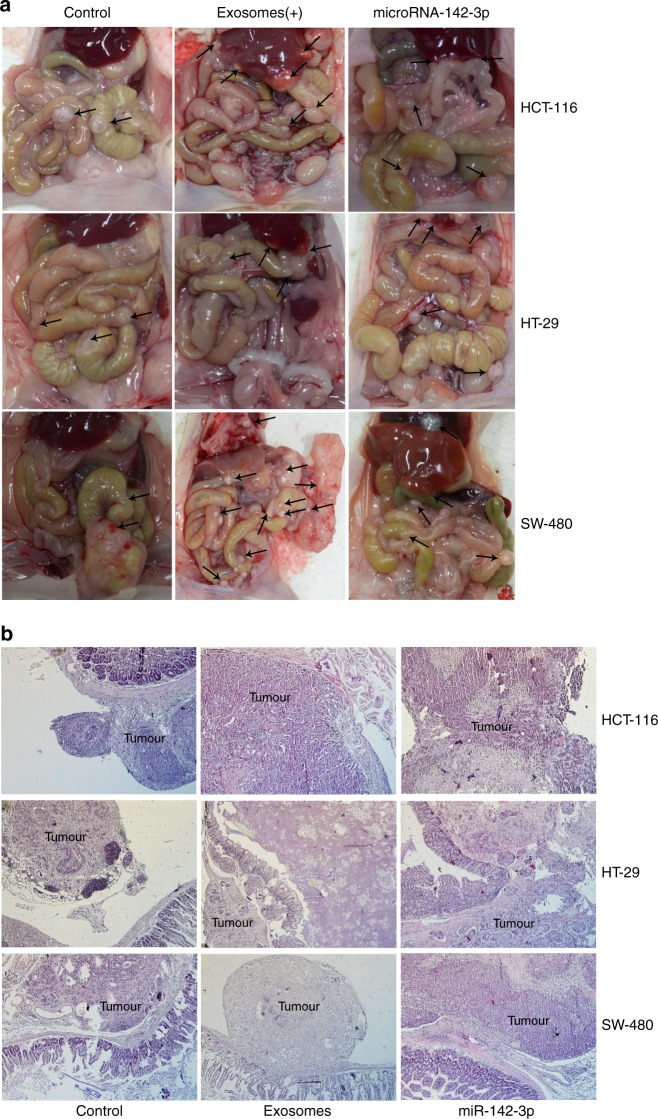
Exosomes from BM-MSCs or miR-142-3p boosted tumourigenesis in colon cancer. **a** Exosomes from BM-MSCs or miR-142-3p boosted tumourigenesis and progression of HCT-116, HT-29 and SW-480 cells in vivo. The black arrows showed the tumours in primary or secondary regions. **b** H&E staining showed primary tumours of HCT-116, HT-29 and SW-480 cells in vivo. The tumour tissues were labelled with “tumour”. ×100

Additionally, miR-142-3p-overexpressed colon cancer cells were also implanted into the intestinal wall of BALB/c Nu/Nu mice. Thirty days after orthotopic injection, we found that miR-142-3p inhibited tumour proliferation in the primary tumour of HT-29 and SW-480 colon cancer cells in vivo (Fig. 5). However, miR-142-3p boosted the tumour progression of the three colon cancer cells, and we analysed the number of mice presenting secondary tumours (tumour metastasis). Tumour take rate is shown in Supplementary Table 2. To further explore the tumourigenesis, we used an in situ tumour formation model and found that the exosomes promoted the formation of secondary tumours (metastases) in colon cancer. However, the exosomes did not influence the tumour weight of the primary tumour in colon cancer (Fig. 5a, Supplementary Table 2). Haematoxylin and Eosin (H&E) staining showed the primary tumours in colon tissues from mice treated with exosomes from BM-MSCs (Fig. 5b). Together, these results suggested that BM-MSC-derived exosomes may contribute to the progression of tumours through miR-142-3p.

### Target genes of exosomal miR-142-3p in colon cancer cells

**Fig. 6:**
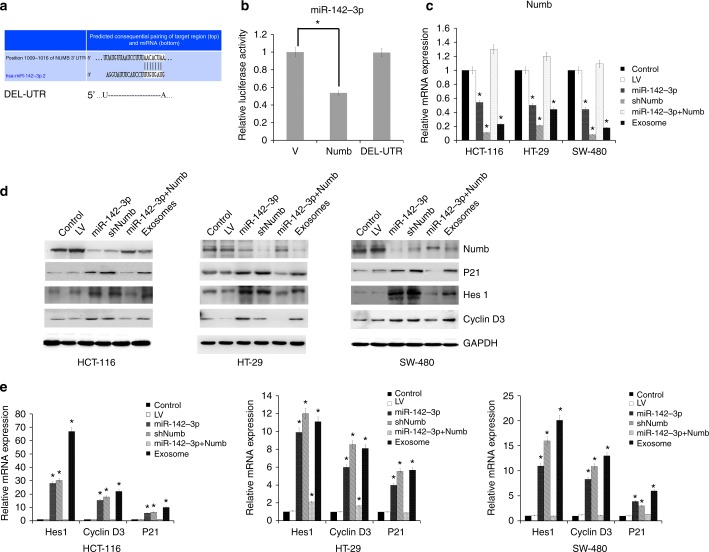
Numb is the target gene of exosomal miR-142-3p in colon cancer cells. **a** Schematic diagram of putative binding sites of miR-142-3p in the 3′UTR of Numb. DEL-UTR denotes deletion. **b** Dual-luciferase reporter assay showed that Numb is the target gene of miR-142-3p in human HCT-116 cells. V negative vector, Numb Numb wild-type vector, DEL-UTR Numb 3′UTR deleted vector. **c** Real-time PCR showed the expression of Numb in colon cancer cells transfected with control Lentivirus or miR-142-3p-Lentivirus. **d** Western blot showed the expression of Numb in colon cancer cells transfected with control Lentivirus or miR-142-3p-Lentivirus. **e** Real-time PCR showed the expression of Notch target gene: Hes1, P21, and cyclin D3 in colon cancer cells transfected with control Lentivirus or miR-142-3p-Lentivirus. *P < 0.05. **f** Western blot showed the expression of Notch target gene: Hes1, P21, and cyclin D3 in colon cancer cells transfected with control Lentivirus or miR-142-3p-Lentivirus

To identify the target of miR-142-3p that mediated the stemness strait in colon cancer cells, we used TargetScan to determine miR-142-3p target genes and identified several target genes related with the CSC signalling pathway. Isobe et al. show that miR-142-3p targets the APC gene in human breast CSCs and inhibits the renewal of stem cells.^21^ However, mutations in the APC gene have been linked to colon cancer, and it has been suggested that the mutations inactivate APC in cancer cells to promote unregulated cell growth.^22–24^ Through bioinformatic analysis of our data, we selected Numb as the target gene of miR-142-3p in colon cancer (Fig. 6a). Using dual-luciferase reporter assay, we found that miR-142-3p could bind directly to the 3′UTR of Numb, while no difference was noted with the mutant 3′UTR (Fig. 6b). How Numb inhibits the signalling activity of Notch has been testified for a long time, which may directly or indirectly inhibit Notch.^25^ Using quantitative real-time PCR, we found that Numb expression in colon cancer cells transfected with miR-142-3p was significantly decreased compared with colon cancer cells transfected with a negative control. Correspondingly, upregulating Numb in miR-142-3p-overexpressed colon cancer cells could reverse the impact on Numb expression. We also detected the expression of Notch target genes in exosomes from BM-MSCs retrospectively. The results showed that exosomes from BM-MSCs gained the similar effect with miR-142-3p-overexpressed colon cancer cells (Fig. 6c). By western blot, we confirmed that the abundance of Numb was decreased in miR-142-3p-transfected colon cancer cells compared with controls. Furthermore, inhibiting the expression of Numb promoted Notch target gene expression, such as Hes1, P21 and cyclin D3. Meanwhile, miR-142-3p and exosomes from BM-MSCs inhibited the expression of Numb and promoted Notch signal. Reversing Numb in miR-142-3p-overexpressed colon cancer cells could also inhibit Notch signal (Fig. 6d).

To further analyse the effect of miR-142-3p and exosomes from BM-MSCs on Notch signal pathway in human colon cancer, we examined the expression of Numb and Notch target genes: Hes1, P21, and cyclin D3, by real-time PCR. The results also showed that miR-142-3p downregulated Numb, and miR-142-3p and exosomes from BM-MSCs inhibited the expression of Numb and promoted Notch signal. Reversing Numb in miR-142-3p-overexpressed colon cancer cells could also inhibit Notch signal, consistent with (Fig. 6d, e).

### Deprived miR-142-3p from exosomes of BM-MSCs weakened the effect of exosomes on colon cancer

To further investigate the role of miR-142-3p on colon CSCs, we tried to identify the effect of exosomal miR-142-3p on colon cancer cells by depriving miR-142-3p from exosomes of BM-MSCs. With antagomiR-142-3p, we decreased the expression of miR-142-3p in the exosomes of BM-MSCs.

We treated the colon cancer cells with exosomes from antagomiR-142-3p-transfected BM-MSCs or control antagomiR-transfected BM-MSCs (Supplementary Fig. S7A). From Fig. S7B, we can see that antagomiR-142-3p transfection markedly inhibited the expression of miR-142-3p in exosomes from antagomiR-142-3p-transfected BM-MSCs, compared with control antagomiR-transfected BM-MSCs. Real-time PCR showed that exosomes from antagomiR-142-3p decreased the transfer of miR-142-3p from exosomes (Supplementary Fig. S7C). We still detected CD133 and Lgr5 by FCM and colony-formation assay. The results showed that antagomiR-142-3p could downregulate the population of colon CSCs (Supplementary Fig. S7D, G). We also found that the antagomiR-142-3p therapy relatively decreased cell invasion and adhesion compared with the control antagomiR (Supplementary Fig. S7E, F). A doxorubicin drug resistance assay showed that antagomiR-142-3p exosomes could significantly inhibit the cell viability of colon cancer cells compared with exosomes from BM-MSCs (Supplementary Fig. S7H). From the results above, we can see that depriving miR-142-3p from exosomes of BM-MSCs could weaken the effect of exosomes on colon cancer cells.

These findings strongly indicate that exosomal transfer of miR-142-3p from BM-MSCs decreased Numb expression in colon cancer cells, activating the Notch signal pathway. Based on these findings, we propose a mechanism involving exosomal miR-142-3p–Numb–Notch signalling axis, which can significantly enhance the population of colon CSCs (Supplementary Fig. S8).

## Discussion

BM-MSC migration to primary tumours and their participation in the tumour microenvironment has been thoroughly understood.^26–28^ The function of exosomes from BM-MSCs has been explored in many areas, such as dermis repair and regeneration,^29^ osteoarthritis,^30^ breast cancer,^18^ multiple myeloma,^9^ etc. However, how BM-MSC-derived exosomes influence tumour stemness is unclear.

To explore the influence of BM-MSC-derived exosomes on colon cancer cells, we cultured BM-MSCs and isolated the exosomes (Figs. 1,  2). We treated colon cancer cells with BM-MSC-derived exosomes 10 μg/ml q.o.d (at days 1, 3, 5, 7…) for 1–2 weeks. The identification (or existence) of CSCs remains a point of controversy. There are no solid markers used to identify CSCs from colon cancer specimens. Here we selected CD133 and Lgr5 as cell surface markers of colon CSCs. We first confirmed that BM-MSC-derived exosomes increased the population of colon cancer cells with cell surface markers by FCM and with properties of CSC functions (Fig. 3). There are clinical implications that the population of CSCs may be the “bad seed” of cancer and resistant to chemotherapy drugs, so it is important to investigate cell invasion, drug resistance states and tumour colony-formation ability. Correspondingly, the expression levels of stem cell-associated genes, including OCT4, Lin28, Lgr5, KLF, Bmi-1, CD44 and SOX2, increased sharply in colon cancer cells upon treatment with exosomes from BM-MSCs or exosomal miR-142-3p (Figs. 3d,  4e). The findings suggest that exosomes from BM-MSCs play a key role in sustaining CSCs. Importantly, the exosomes promoted tumourigenesis in orthotopic tumour growth mice by subcutaneous model or in situ model (Supplementary Fig. S6 and Fig. 5). To explore the underlying mechanism of this process and find out which role the exosomes play in the process, we analysed the microRNA array (Supplementary Fig. S1, 2) and identified 50 miRs (Supplementary Fig. S3). Among them, we detected the microRNA that could promote the population of CSCs by mimic transfection and selected miR-142-3p (Supplementary Fig. S3, 4). The results showed that the miR-142-3p could prominently upregulate the population of colon CSCs (Fig. 4). We determined that exosomal miR-142-3p is one of the important microRNAs through which the colon CSC population is enriched in colon cancer by the microenvironment. In short, miR-142-3p is the main factor increasing the colon CSC population in colon cancer. Many researchers found that miR-142-3p inhibited tumour proliferation and invasion as an suppressor.^31–33^ Our research also found that miR-142-3p could inhibit the proliferation of colon cancer cells. However, different from their views, we showed that miR-142-3p promoted the population of CSCs in colon cancer as a tumour booster. Our view is supported by Isobe T and Li Y et al.^21,34^ However, the viewpoint of Shen and colleagues is different from ours^35^; the authors showed that miR-142-3p inhibited colon CSCs by targeting CD133 and Lgr5 in SW116 and Caco2. Although they showed that CD133 and LGR5 were targets of miR-142-3p, we did not find CD133 and LGR5 to be downregulated by miR-142-3p in the cells used in the current study (Supplementary Fig. S9). It may be that some underlying mechanism is yet to be revealed that will explain these apparently different results. Many questions still remain around the role and mechanistic action of miR-142 in cancer.

The observation that CSCs are regulated and supported by BM-MSCs has been reported by several groups.^36,37^ They have showed that MSCs traffic from bone marrow to primary tumour sites in xenografts and explained the mechanism by the cytokine network. In our study, we demonstrated that BM-MSCs produce exosomes containing miR-142-3p into colon cancer cells to promote their CSC phenotype. Our findings suggest that BM-MSC-derived exosomes play an important role in establishing a niche for CSCs through exosomal miR-142-3p. We demonstrated here that Numb, an inhibitor of the Notch signalling pathway, is a target of miR-142-3p. MiR-142-3p inhibits the expression of Numb and promotes the expression of Notch target genes, such as Hes1, P21 and Cyclin D3 (Fig. 6).

In summary, our work sheds light on an area of contention in the ability of BM-MSC-derived exosomes to modulate colon cancer progression. These data elucidate the function of BM-MSC-derived exosomes in promoting the CSC phenotype via miR-142-3p. Our findings, along with other studies in the field, suggest that BM-MSCs, the more versatile cells, contribute to the colon CSCs phenotype, tumour formation, invasion and chemotherapy resistance through cytokines or exosomes. Given that BM-MSC-derived exosomes could increase the population of colon CSCs in colon cancer, it would thus be improper to treat colon cancer patients with BM-MSCs or exosomes from BM-MSCs.

## Electronic supplementary material

suppl1

suppl2

## Availability of data and materials

The datasets used and analysed during the current study are available from the corresponding author on reasonable request.
