# Supplementary material for: Exosomes from BM-MSCs increase the population of CSCs via transfer of miR-142-3p
Source: Br J Cancer. 2018 Sep 17;119(6):744–55. doi: 10.1038/s41416-018-0254-z (PMC6173771; doi:10.1038/s41416-018-0254-z)
Supplement: Supplementary file 1 — suppl1 [file 41416_2018_254_MOESM1_ESM.docx]

Table 1, Primers

| Numb | F:TCAGCAGATGGACTCAGAGTT |
| --- | --- |
|  | R:AGGCTCTATCAAAGTTCCTGTCT |
| Actin | F:CATGTACGTTGCTATCCAGGC |
|  | R:CTCCTTAATGTCACGCACGAT |
| GAPDH | F:TGTGGGCATCAATGGATTTGG |
|  | R:ACACCATGTATTCCGGGTCAAT |
| P21 | F:GCAGACCAGCATGACAGATTT |
|  | R:GGATTAGGGCTTCCTCTTGGA |
| Cyclin D3 | F:TACCCGCCATCCATGATCG |
|  | R:AGGCAGTCCACTTCAGTGC |
| CD44 | F:TGGCACCCGCTATGTCCAG |
|  | R:GTAGCAGGGATTCTGTCTG |
| Oct-4 | F: GAGAACCGAGTGAGAGGCAACC |
|  | R: CATAGTCGCTGCTTGATCGCTTG |
| SOX2 | F: CCCTGCTGAGAATAGGACAT |
|  | R: CCCTGCAGTACAACTCTATG |
| KLF | F: ACCAGGCACTACCGTAAACACA |
|  | R: GGTCCGACCTGGAAAATGCT |
| Lin28 | F: GGTGCACAAAGACATCCA |
|  | R: GGCATCTGTAAGTGGTTCA |
| Lgr5 | F: TGCTGGCTGGTGTGGATGCG |
|  | R: GCCAGCAGGGCACAGAGCAA |
| Bmi1 | F: ACTTCATTGATGCCACAACC |
|  | R: CAGAAGGATGAGCTGCATAA |
| Hes 1 | F: TCAACACGACACCGGATAAAC |
|  | R: GCCGCGAGCTATCTTTCTTCA |

Table 2, colon cancer cells were injected into intestinal wall of mice in each groups

| **Condition** | **Animal inoculated** | **Animals presenting tumors(metastasis)** | **Tumor take rate(%)** | **Primary tumor size(cm^3^)** |
| --- | --- | --- | --- | --- |
| **HCT-116** | **8** | **1** | **12.50%** | **1.18±0.83** |
| **116+exosomes** | **8** | **7** | **87.50%** | **1.11±0.85** |
| **116+miR142-3p** | **8** | **7** | **87.50%** | **1.16±0.69** |
| **HT-29** | **8** | **0** | **0.00%** | **0.72±0.59** |
| **29+exosomes** | **8** | **7** | **87.50%** | **0.78±0.60** |
| **29+miR142-3p** | **8** | **7** | **87.50%** | **0.53±0.49** |
| **SW-480** | **8** | **1** | **12.50%** | **1.03±0.72** |
| **480+exosomes** | **8** | **8** | **100.00%** | **1.13±0.86** |
| **480+miR142-3p** | **8** | **7** | **87.50%** | **0.55±0.64** |

Number of mice inoculated, mice presenting tumors, tumor take rate and primary tumor size.
