# Supplementary material for: Exosomes from BM-MSCs increase the population of CSCs via transfer of miR-142-3p
Source: Br J Cancer. 2018 Sep 17;119(6):744–55. doi: 10.1038/s41416-018-0254-z (PMC6173771; doi:10.1038/s41416-018-0254-z)
Supplement: Supplementary file 2 — suppl2 [file 41416_2018_254_MOESM2_ESM.pdf]

Fig. S1

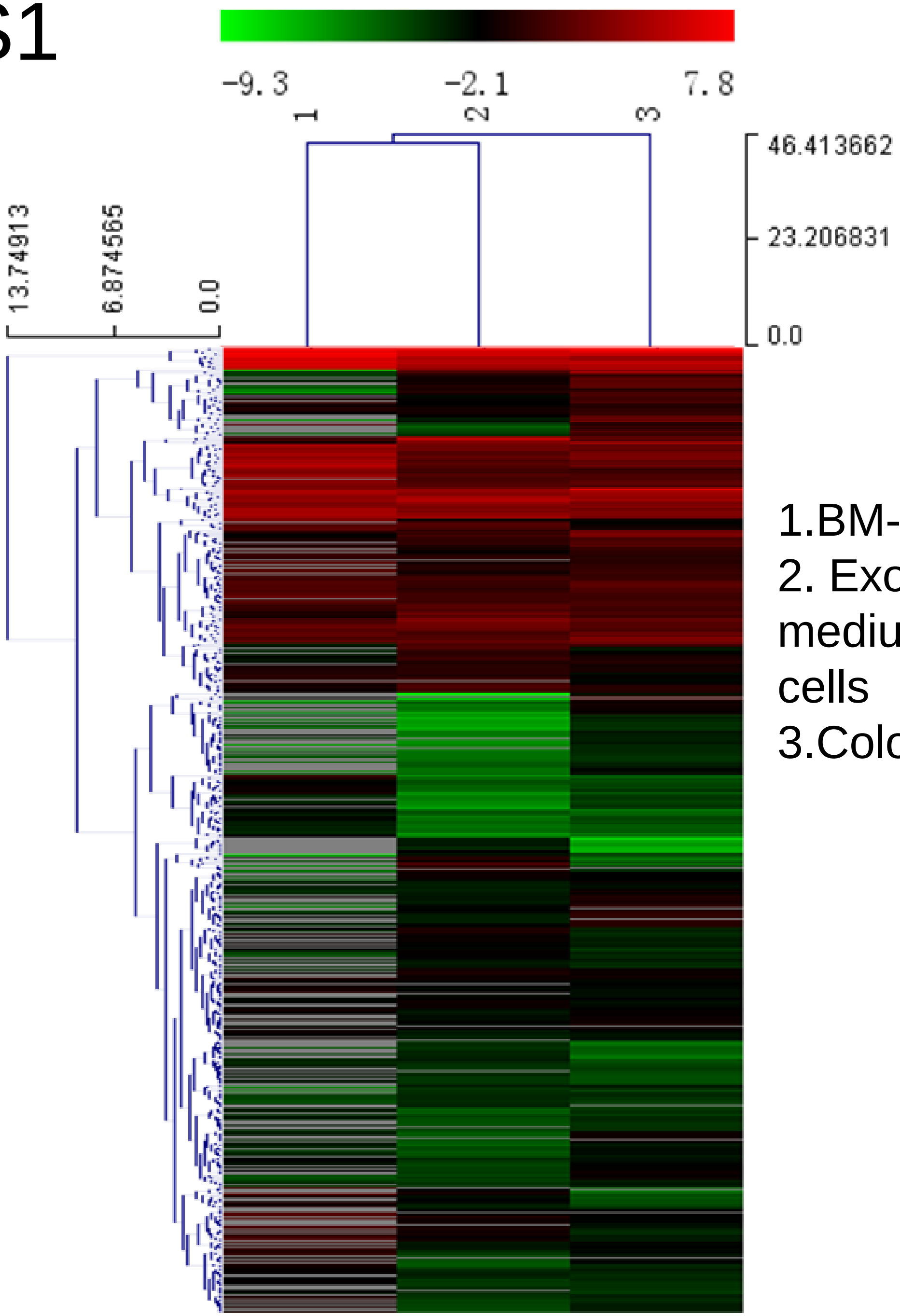


1.BM-MSCs derived exosomes
2. Exosomes derived from co-cultured medium of BM-MSCs and colon cancer cells
3.Colon cancer cells derived exosomes

# Fig. S2

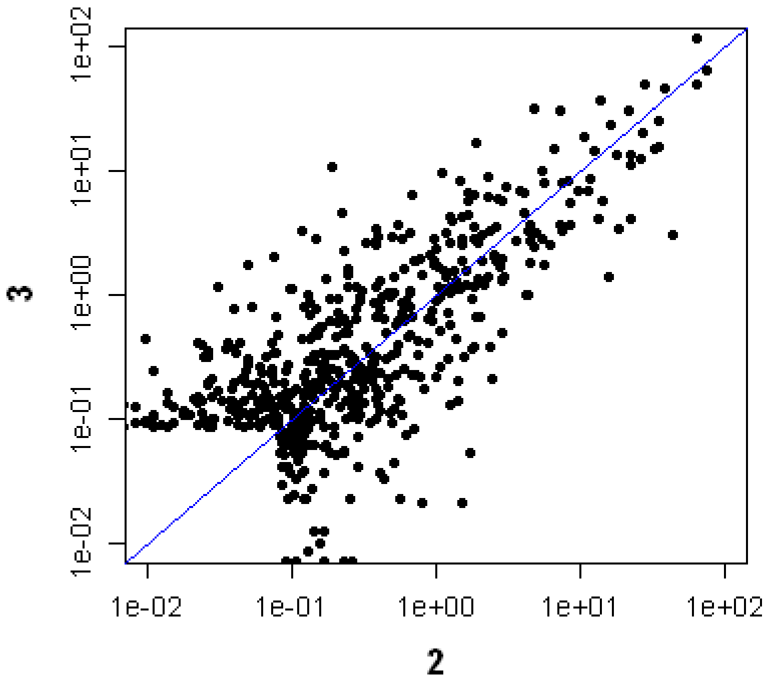


Correlation coefficient matrix

| | 2 | 3 |
|---|---|---|
| 2 | 1 | 0.854486 |
| 3 | 0.854486 | 1 |

2. Exosomes derived from co-cultured medium of BM-MSCs and colon cancer cells
3.Colon cancer cells derived exosomes

# Fig. S3

| miRNAs | Fold change | *P* |
|---|---|---|
| hsa-miR-513c-3p | 32.43121547 | <0.001 |
| hsa-miR-98-3p | 15.5359116 | <0.001 |
| hsa-miR-3651 | 15.05041436 | <0.001 |
| hsa-miR-5008-3p | 14.95331492 | <0.001 |
| hsa-miR-451a | 13.82808814 | <0.001 |
| hsa-miR-935 | 13.25618544 | <0.001 |
| hsa-miR-3648 | 13.16236955 | <0.001 |
| hsa-miR-548e-3p | 12.81712707 | <0.001 |
| hsa-miR-5193 | 11.6832561 | <0.001 |
| hsa-miR-627-5p | 11.47831217 | <0.001 |
| hsa-miR-4417 | 11.00460405 | <0.001 |
| hsa-miR-3201 | 11.00167937 | <0.001 |
| hsa-miR-345-3p | 10.97970676 | <0.001 |
| hsa-miR-142-3p | 9.501128735 | <0.001 |
| hsa-let-7a-2-3p | 8.67198514 | <0.001 |
| hsa-miR-126-3p | 8.262613364 | <0.001 |
| hsa-miR-5572 | 6.980295947 | <0.001 |
| hsa-miR-424-5p | 6.964374167 | <0.001 |
| hsa-miR-1184 | 6.159293312 | <0.001 |
| hsa-miR-4434 | 5.738930931 | <0.001 |
| hsa-miR-651-3p | 5.522531077 | <0.001 |
| hsa-miR-1275 | 5.371155294 | <0.001 |
| hsa-miR-3611 | 5.341075223 | <0.001 |
| hsa-miR-4503 | 5.340469613 | <0.001 |
| hsa-miR-5701 | 5.340469613 | <0.001 |
| hsa-miR-5187-3p | 5.173806382 | <0.001 |
| hsa-miR-3195 | 5.16160221 | <0.001 |
| hsa-miR-3146 | 4.867804902 | <0.001 |
| hsa-miR-3621 | 4.796785441 | <0.001 |
| hsa-miR-4431 | 4.768835769 | <0.001 |
| hsa-miR-4258 | 4.765685527 | <0.001 |
| hsv2-miR-H7-3p | 4.564935065 | <0.001 |
| hsa-miR-3162-3p | 4.556204845 | <0.001 |
| hsa-miR-3915 | 4.352209945 | <0.001 |
| hsa-miR-3687 | 4.283799155 | <0.001 |
| hsa-miR-4632-3p | 4.175276243 | <0.001 |
| hsa-miR-31-3p | 4.132950843 | <0.001 |
| hsa-miR-4648 | 4.126726519 | <0.001 |
| hsa-miR-1915-3p | 3.992967076 | <0.001 |
| hsa-miR-663a | 3.938172941 | <0.001 |
| hsa-miR-223-3p | 3.922056115 | <0.001 |
| hsa-miR-1470 | 3.910949969 | <0.001 |
| hsa-miR-874-5p | 3.803061694 | <0.001 |
| hsa-miR-3654 | 3.722145488 | <0.001 |
| hsa-miR-4284 | 3.623113713 | <0.001 |
| hsa-miR-31-5p | 3.541640105 | <0.001 |
| hsa-miR-485-3p | 3.464088398 | <0.001 |
| hsa-miR-620 | 3.457687643 | <0.001 |

# Fig. S4

| | Exosomes from BMMSC | Exosomes from co-cultured cells | Exosomes from colon cancer cells |
|---|---|---|---|
| 1.microRNA | high+/red | Higher +++ | low+/- green |
| 2.miR-142-3p Array results | 4.0103 | 9.5011 | 1 |
| 3.miR-142-3p qPCR results | 66136.11227 | 236182.8842 | 1 |

# Fig. S5

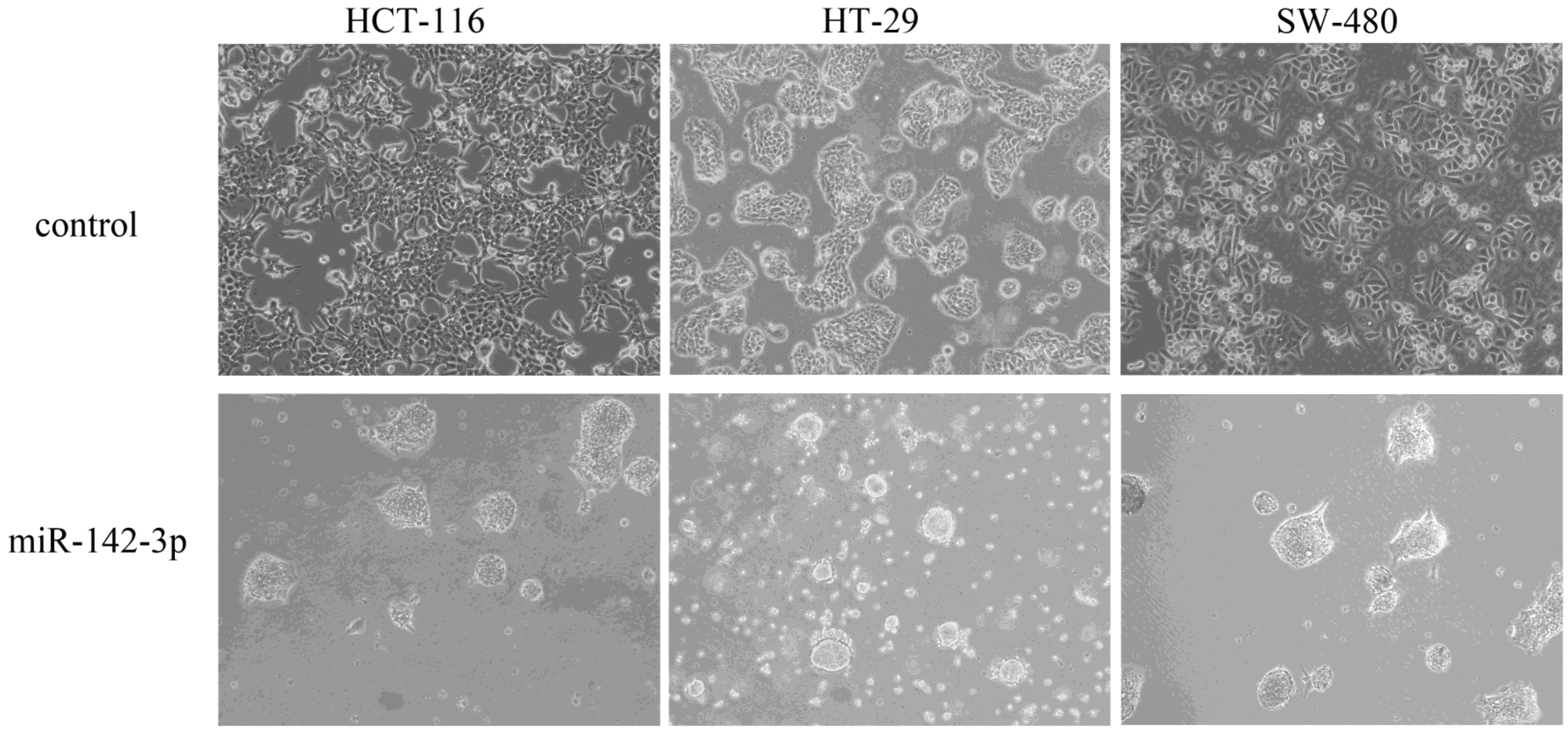

Fig. S6

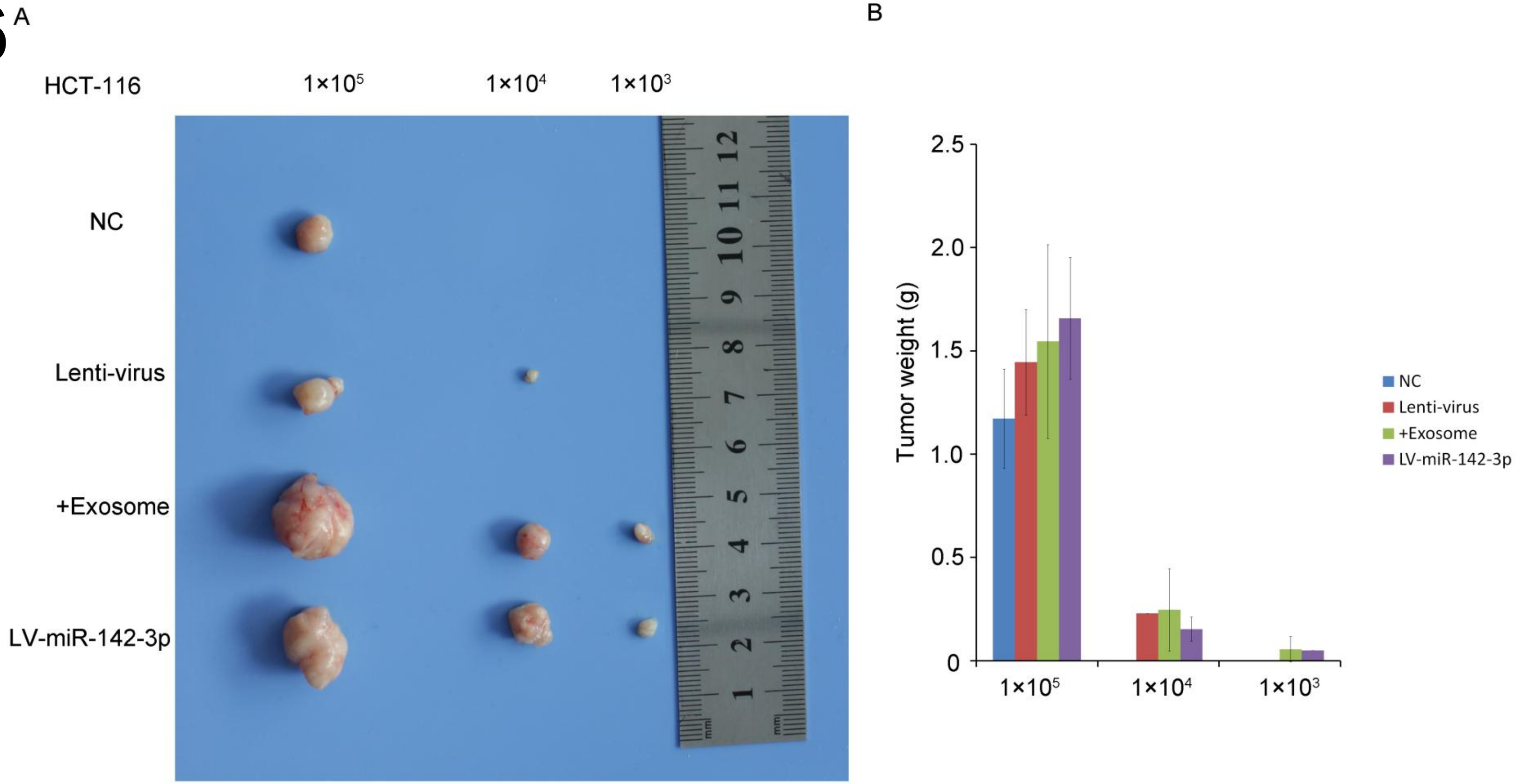


C

| | Cell number inoculated | | |
|---|---|---|---|
| | $1\times10^5$ | $1\times10^4$ | $1\times10^3$ |
| **NC** | **4/5** | **0/5** | **0/5** |
| **Lenti-virus** | **5/5** | **1/5** | **0/5** |
| **+Exosome** | **5/5** | **3/5** | **2/5** |
| **LV-miR-142-3p** | **5/5** | **3/5** | **1/5** |

Fig. S7

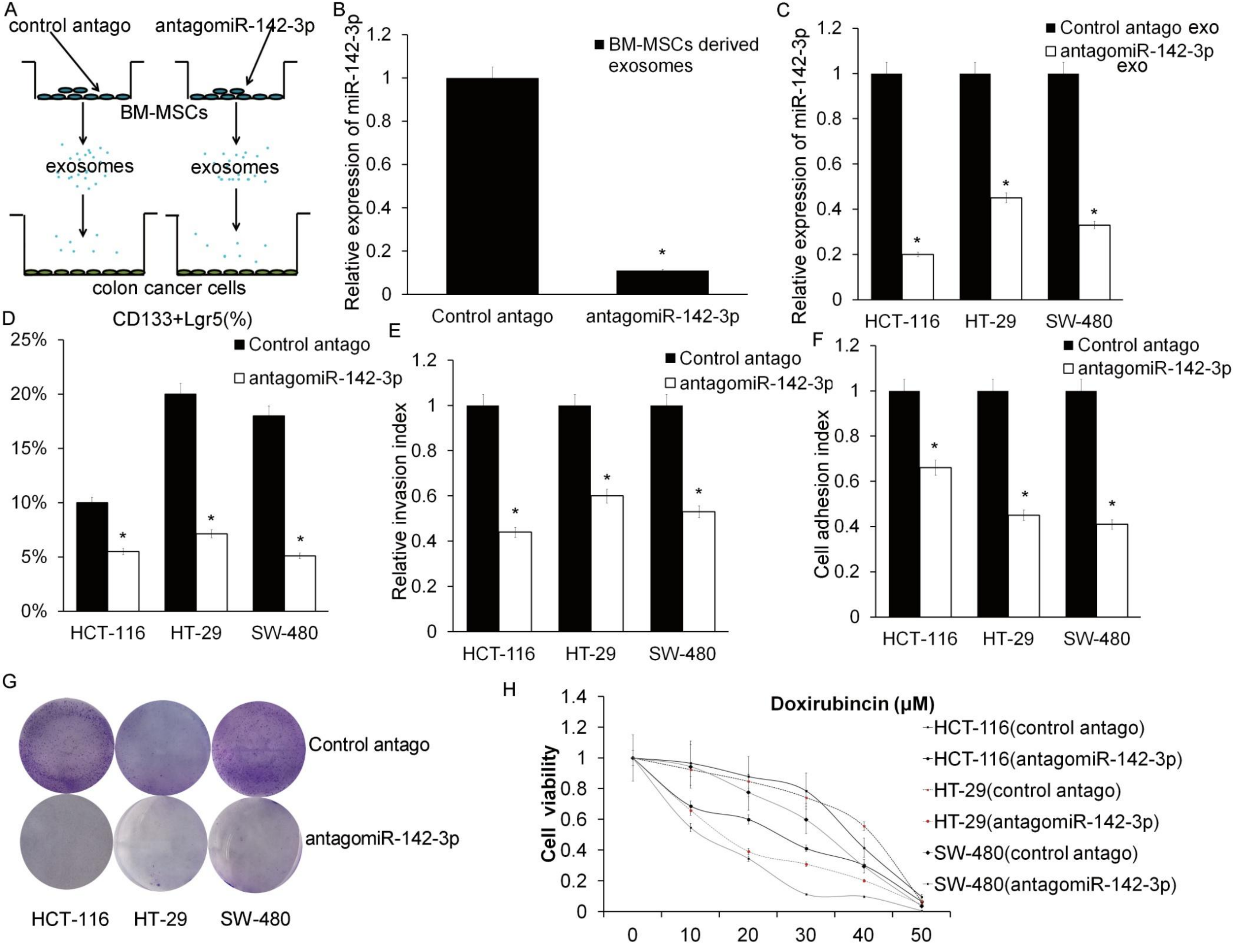

A
control antago
antagomiR-142-3p
BM-MSCs
exosomes
exosomes
colon cancer cells
B
Relative expression of miR-142-3p
BM-MSCs derived exosomes
Control antago
antagomiR-142-3p
C
Relative expression of miR-142-3p
Control antago exo
antagomiR-142-3p exo
HCT-116
HT-29
SW-480
D
CD133+Lgr5(%)
Control antago
antagomiR-142-3p
HCT-116
HT-29
SW-480
E
Relative invasion index
Control antago
antagomiR-142-3p
HCT-116
HT-29
SW-480
F
Cell adhesion index
Control antago
antagomiR-142-3p
HCT-116
HT-29
SW-480
G
Control antago
antagomiR-142-3p
HCT-116
HT-29
SW-480
H
Doxirubincin (μM)
Cell viability
HCT-116(control antago)
HCT-116(antagomiR-142-3p)
HT-29(control antago)
HT-29(antagomiR-142-3p)
SW-480(control antago)
SW-480(antagomiR-142-3p)

# Fig. S8

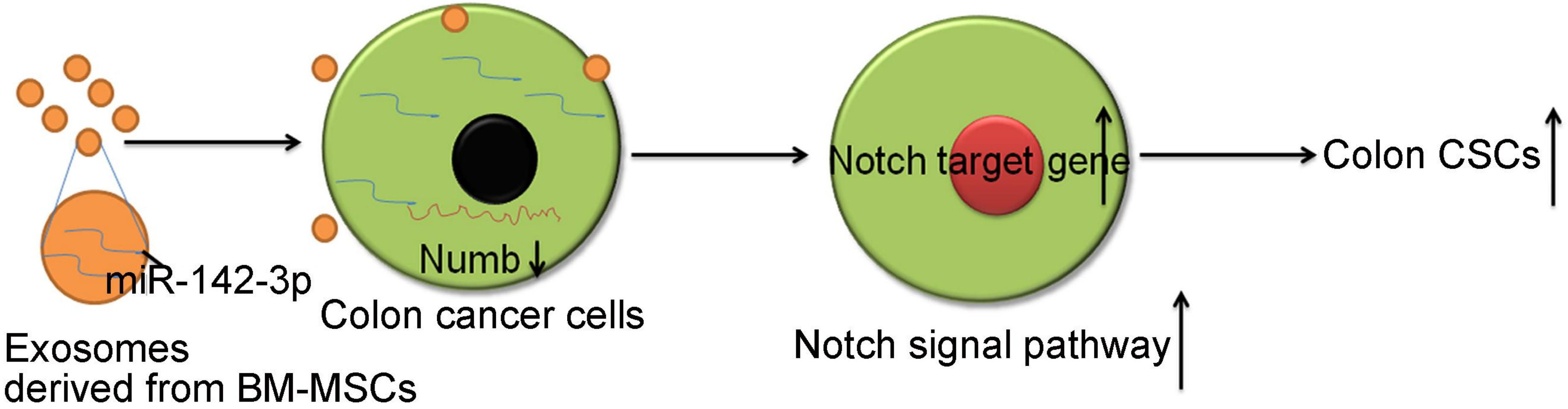


**Fig.8 A cartoon summarizing the results.**
Exosomal transfer of BM-MSCs derived miR-142-3p decreased Numb expression in colon cancer cells.

# Fig. S9

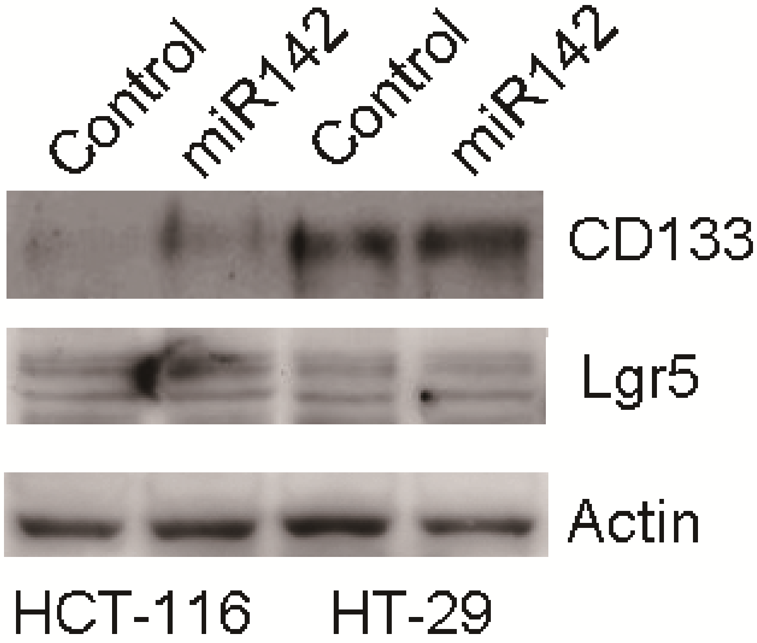


Fig.S9  Western blot showed miR-142 do not inhibit the expression of CD133 and Lgr5 sharply in HCT-116 and HT-29 cells.
